# Supplementary material for: Comparing a Whole Grain Blend with Polished White Rice for Starch Digestibility and Gut Microbiota Fermentation in Diabetic Patients: An In Vitro Study
Source: Foods. 2025 Jul 22;14(15):2557. doi: 10.3390/foods14152557 (PMC12345654; doi:10.3390/foods14152557)
Supplement: Supplementary file 1 [file foods-14-02557-s001.zip › foods-3747102-supplementary.pdf]

Supplementary Table S1. Starch hydrolysis rates (%) of WR and WGB during *in vitro* digestion and statistical analysis by repeated measures ANOVA.

Time (min)	WR (mean ± SD, %)	WGB (mean ± SD, %)	Group Effect	Time Effect	Time × Group interaction effects)
**30**	5.81±1.41	5.42±2.90	***F***=3.32	***F***=124.02	***F***=0.679
**60**	5.13±0.95	5.79±3.06	***P*** =0.078	***P*** < 0.001	***P*** =0.688
**90**	5.24±0.48	6.23±4.49			
**120**	10.38±2.12	5.44±3.17			
**150**	44.44±6.81	42.96±5.35			
**180**	65.05±15.11	56.61±2.73			
**210**	78.00±14.38	66.10±10.19			
**240**	73.02±6.16	69.76±5.71			

Values are presented as mean ± SD. Statistical significance was assessed using repeated-measures ANOVA with time, group, and interaction effects. WR: polished white rice; WGB: whole grain blend
